# Preparation, Structural Characterization of Anti-Cancer Drugs-Mediated Self-Assembly from the Pluronic Copolymers through Synchrotron SAXS Investigation

**DOI:** 10.3390/ma15155387

**Published:** 2022-08-05

**Authors:** Tz-Feng Lin, Wei-Chieh Wang, Xin-Yu Zeng, Yi-Xian Lu, Pei-Jung Shih

**Affiliations:** 1 Department of Fiber and Composite Materials, Feng Chia University, Taichung 407, Taiwan; 2 Master’s Program of Biomedical Informatics and Biomedical Engineering, Feng Chia University, Taichung 407, Taiwan

**Keywords:** drug carrier, self-assembly, micelle, lamellar, hydrogel, microenvironment, biomedical composite

## Abstract

Chemotherapy drugs are mainly administered via intravenous injection or oral administration in a very a high dosage. If there is a targeted drug vehicle which can be deployed on the tumor, the medical treatment is specific and precise. Binary mixing of biocompatible Pluronic^®^ F127 and Pluronic^®^ L121 was used in this study for a drug carrier of pluronic biomedical hydrogels (PBHs). Based on the same PBH ingredients, the addition of fluorouracil (5-FU) was separated in three ways when it was incorporated with pluronics: F127-L121-(5-FU), F127-(5-FU), and L121-(5-FU). Small angle X-ray scattering experiments were performed to uncover the self-assembled structures of the PBHs. Meanwhile, the expected micelle and lamellar structural changes affected by the distribution of 5-FU were discussed with respect to the corresponding drug release monitoring. PBH-all with the mixing method of F127-L121-(5-FU) has the fastest drug release rate owing to the undulated amphiphilic boundary. In contrast, PBH-2 with the mixing method of L121-(5-FU) has a prolonged drug release rate at 67% for one month of the continuous drug release experiment because the flat lamellar amphiphilic boundary of PBH-2 drags the migration of 5-FU from the hydrophobic core. Therefore, the PBHs developed in the study possess great potential for targeted delivery and successfully served as a microenvironment model to elucidate the diffusion pathway of 5-FU.

## 1. Introduction

Lipid nanoparticles has clinically emerged as potent and flexible platform for the therapeutical COVID vaccines [1,2]. This advanced drug delivery system owns tailored hydrophilic and hydrophobic segments/blocks and the overall structure gives low cytotoxicity and immunogenicity. Synthetic triblock copolymers of pluronics are one of the most famous examples and provide exciting opportunities in precision medicine applications [3]. Pluronic^®^ F127 (i.e., F127) has attracted significant interest in the last two decades, and has achieved tremendous clinical success since the approval of thermosensitive injectable smart hydrogels for drug delivery [4]. In fact, using F127 as a novel soft template extends its applications to biological theragnostic [5], mesoporous silica film [6], mesoporous silica aerogels [7] and mesoporous metal–organic frameworks [8], as well as in other varied sciences. In addition, small angle X-ray scattering (SAXS) studies have demonstrated micelles of F127 [9] that integrate into a face-centered cubic (FCC) structure [10]. In an assessment of lamellar-forming Pluronic^®^ L121 (i.e., L121) [11] in the drug delivery system, it was considered to have a higher solubilization capacity of poorly water soluble drugs than spherical micelles [12]. However, the lack of structural stability in aqueous dispersion was the result of a steric stabilizing effect [13]. Binary mixing of hydrophobic and hydrophilic Pluronic copolymers was proposed synergistically to overcome the steric stabilizing effect and produced a stable drug delivery vehicle for anti-cancer drugs [14]. Systems have been well studied such P85/P123 [15], P407/F127 and P188/F68 [16], L64/F127 [17], L81/F127 [18], L121/F127 [19,20] and (P123/F127)/(L61, L81, L101, and L121) [21]. The use of curcumin [21], doxorubicin [22], paclitaxel [23], fluorouracil (5-FU) [24] and others have also been tried in binary pluronic-based hydrogels for controlled release. Although their physicochemical property, drug loading, encapsulation efficiency and cellular uptake in biochemistry are not only well known but also effective in eliminating animal or human tumors, nevertheless, a high dosage is generally required in intravenous injection or oral administration that would cause side effects to patients. If effective drug release is sustained for a very long time around a local tumor, it could improve the quality of the patient’s recovery. Moreover, the prolonged drug release up to one month from the binary mixing of pluronic copolymers with 5-FU has rarely been seen in the literature or discussed. Information in drug migration or diffusion pathways from drug delivery vehicles is needed for cancer chemotherapy. In this study, binary mixing of pluronic copolymers using F127 and L121 was established as a microenvironment model. The anti-cancer drug, 5-FU, widely used for patients in the treatment of colon, breast and other cancers was deployed in the microenvironment model to control the drug release. Experiments followed similar preparation methods with the same ingredients as pluronic biomedical hydrogels (PBHs). The novelty and relevance of the work is in differentiating the delivery pathway of 5-FU in PBHs, which controls the drug releasing rate. The incorporation of 5-FU with binary pluronic copolymers and how the preparation sequence may affect the structural changes in micelles and lamellae was revealed in the release profiles. Detailed characterization was provided by small angle X-ray scattering and then a molecular concept was highlighted. A distinct drug release pathway was thereafter deduced with a variation in the internal structure to fulfill a microenvironment model. Understanding of the microenvironment model could help to rationalize adjustments in the microstructure of PBHs to preserve anti-cancer drug persistence.

## 2. Materials and Methods

Pluronic^®^ triblock copolymers of F127 (PEO_100_-PPO_65_-PEO_100_) and L121 (PEO_5_-PPO_68_-PEO_5_) have an average Mn at ~12,600 g/mol and ~4400 g/mol, respectively. They are designated by their nomenclature indexes, e.g., ‘F127’ means ‘Pluronic^®^ F127’, and L121 as well. F127, L121, Tween 20, and dimethyl sulfoxide (DMSO) were purchased from Sigma-Aldrich and used as received without further purification. All chemicals used were of analytical grade. 5-FU was used as an anti-cancer drug and was purchased from AdooQ^®^ Bioscience and stored in DMSO as 0.2 M at 4 °C. The pluronic biomedical hydrogels (PBHs) used in this study are presented in Table 1. The binary mixing of F127 and L121 was proceeded by the ratio of volume 100:300. Ingredients in the PBH were all mixed together at the same time via sonification. For the sample code PBH-1, the ingredients of F127 and 5-FU were first mixed to make the 5-FU encapsulation in the F127. The mixture was finally mixed with L121 for further study. In contrast, 5-FU was encapsulated in the L121 first and then was mixed together with F127 as PBH-2 for the following characterization studies.

To measure the amounts of 5-FU medicine during drug release experiments, PBHs were placed in an Eppendorf tube, and 600 μL PBS (pH 7.4, containing 0.1% Tween 20 (*v*/*v*)) was pipetted onto the upper part of the Eppendorf tube. These as-prepared Eppendorf tubes were stored in a 37 °C sink under agitation at 100 rpm for the monitoring of 5-FU drug release. In each time set of the measurement, the amount of drug-released 5-FU was determined at OD265 using a calibration curve generated from known concentrations of 5-FU. Fresh PBS was replaced each time for further analysis. Triplicate experiments were performed to measure the optical absorbance average with respect to the time evolution. PBHs without 5-FU were conducted under the same conditions as controls.

SAXS measurements were recorded at the TLS BL23A beamline of the National Syn-chrotron Radiation Research Center (NSRRC), Hsinchu, Taiwan. The X-ray passed through a pinhole with a diameter of 0.5 mm. The incident X-ray beam was focused vertically using a mirror and mono-chromated to the photon energy of 15 keV using double Mo/B_4_C multilayer monochromators. The wavelength of the X-rays was 0.82654 Å. The sample-to-detector distance was 3331.400 mm to give a q* range of 0.005–0.5 Å^−1^, where q* is the scattering vector, related to the scattering angle (2θ) and the photon wavelength (λ) by *q* = 4π sin(θ)/λ = 2π/d, θ is the Bragg angle, and d is the average distance of scattering. The detector was a Pilatus 1M-F area detector with an effective size of 169 mm by 179 mm and a pixel size of 172 μm by 172 μm. The liquid samples were sealed in the sample cell with Kapton-walled windows, and the sample thickness was 2.5 mm. The samples were kept at 25 °C during the SAXS measurements using a thermostatically controlled circulation bath. The collected 2D data were circularly averaged to obtain the 1D scattering intensity distribution as a function of the scattering vector. The measured scattering intensities were corrected for the detector sensitivity and background and normalized to obtain the absolute scattering cross sections using standard HDPE or pure water [25]. The XRD patterns were measured at beamline TLS 17B in NSRRC, Hsinchu City, Taiwan.

## 3. Results

### 3.1. Preparation of Pluronic Biomedical Hydrogels

**Figure 1:**
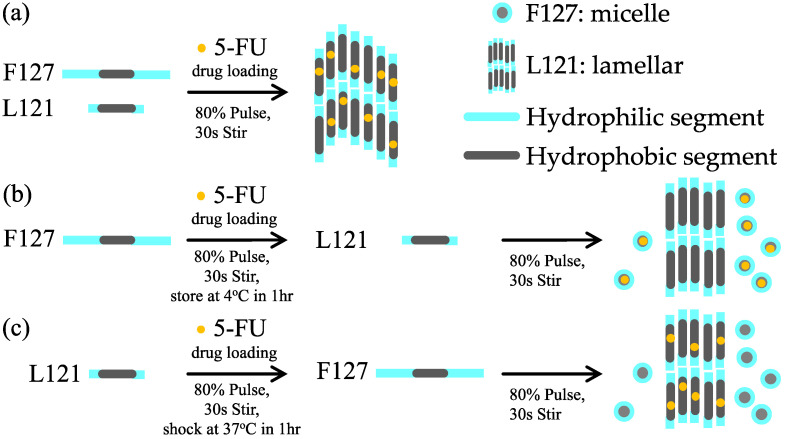
Illustration of sample preparation of the pluronic biomedical hydrogels. The encapsulation of 5-FU is presented as a yellow dot. (**a**) PBH-all; (**b**) PBH-1, and (**c**) PBH-2.

In some Pluronic^®^ triblock copolymers, their self-assembled structure was not stable after preparation, revealing formation of large aggregates or phase precipitation within one week. Therefore, binary mixing of Pluronic^®^ triblock copolymers was suggested to stabilize aqueous dispersions of the self-assembled structure [13]. The experiment design used for preparation of PBHs is presented in Figure 1. The one-step preparation process of 5-FU drug-loaded in the binary mixing F127/L121 was named as PBH-all. F127 and L121 in distilled water were mixed in the proportions indicated. After the addition of 5-FU, the binary mixture was stirred sonically for 30 s. Another two binary mixes, PBH-1 and PBH-2, were prepared using two steps. First, F127 and L121 were individually sonicated with 5-FU via an 80% pulse for 30 s. The mixture solutions were then transferred to 4 °C and 37 °C for one hour, respectively. When the 5-FU was able to be inserted into the single pluronic triblock copolymer, the corresponding L121 and F127 was stirred sonically for 30 s for PBH-1 and PBH-2. The above obtained binary mixtures were characterized as described below.

### 3.2. Self-Assembly and Characteristics of the Pluronic Biomedical Hydrogels

**Figure 2:**
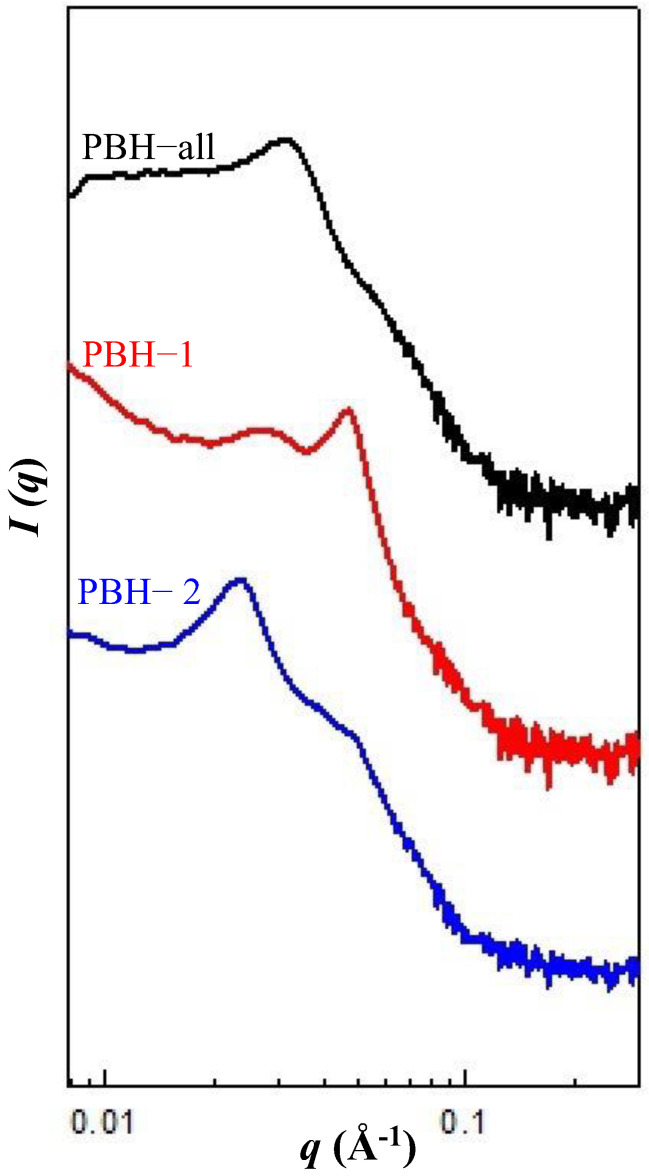
SAXS 1D profiles (offset for clarity, arbitrary intensity units) of the pluronic biomedical hydrogels at 25 °C.

**Figure 3:**
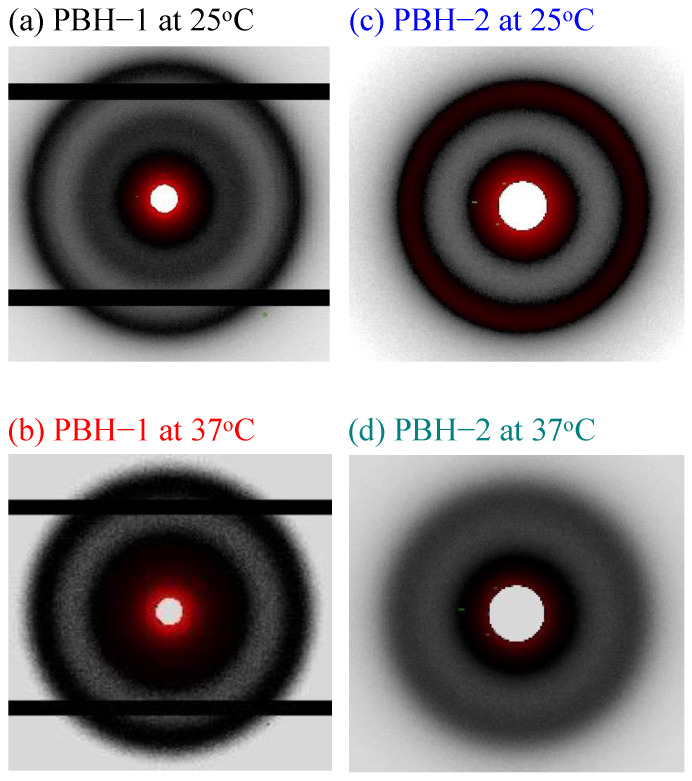
2D-SAXS of the pluronic biomedical hydrogels at 25 °C and 37 °C, respectively.

**Figure 4:**
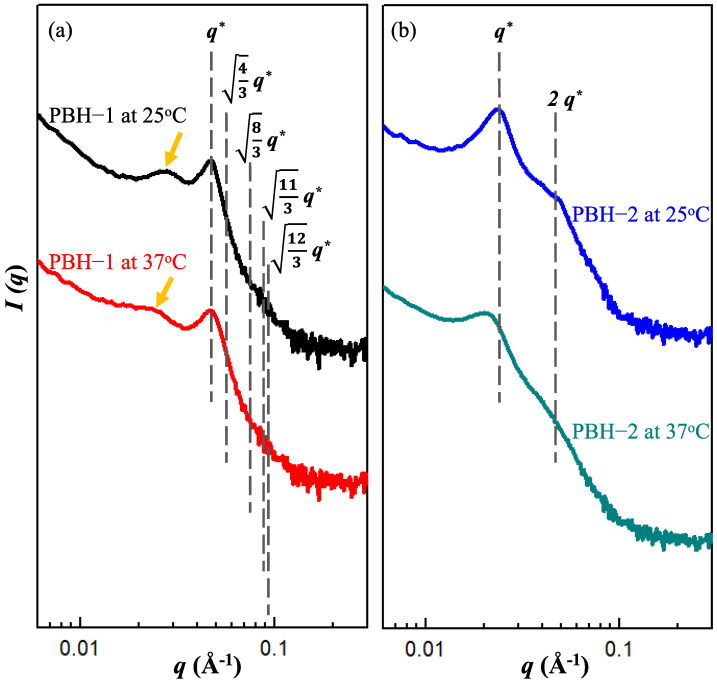
SAXS 1D profiles (offset for clarity, arbitrary intensity units) of the pluronic biomedical hydrogels (**a**) PBH-1 and (**b**) PBH-2, respectively at 25 °C and 37 °C. The black dotted lines serve as a guide for the eyes and the yellow arrow indicates the scattering peak of aggregated micelles.

The stable aqueous dispersions of the PBHs were obtained over half a year after preparation. In Figure 2, well defined scattering peaks can be observed for all PBHs by taking advantage of the self-assembly features of amphiphiles. For PBH-all, a primary scattering peak at q* = 0.033 Å^−1^ was noted while two small shoulders were barely observed around q* = 0.04 and 0.053~0.067 Å^−1^. The suggested structure is a loose packed micellar structure due to the co-assembly of F127 and L121 [26]. For PBH-1, a relative sharp scattering peak was noted at q* = 0.046 Å^−1^ and a broad scattering peak was observed at q* = 0.029 Å^−1^. The scattering peak at q* = 0.046 Å^−1^ may suggest an FCC structure from F127 but is missing a long-range order after the addition of 5-FU. The scattering peak at q* = 0.029 Å^−1^ along with the upward scattering at the low q* area evidenced the aggregation behavior of PBH-1 at an average size of around 22 nm. For PBH-2, there was a clear primary scattering peak at q* = 0.023 Å^−1^ and a secondary scattering peak at q* = 0.046 Å^−1^. According to the scattering q* relative positions of 1:2, 3~5 layers of lamellar structure were identified. In order to further confirm the structural aggregation, PBH-1 and PBH-2 were raised to 37 °C. An isotropic azimural scattering in the 2D-SAXS pattern is illustrated in Figure 3 before and after the temperature changes. An analysis of the one-dimensional profile is presented in Figure 4. The yellow arrow in Figure 4a indicates the scattering peak of aggregated micelles. PBH-1 originally behaves as a weak FCC structure with aggregated micelles in the aqueous media. Lamellar forming of PBH-2 is stronger at 25 °C, however, the scattering intensity is a little bit lower at 37 °C showing the deterioration of the lamellar structure upon micelle dissociation [27]. Furthermore, the scattering peak set 1:2 shifts at the low q*, which means a larger lamellar structure upon thermal expansion of the molecular chain. This may facilitate the expulsion of 5-FU from the lamellar microstructure. Phase transition from lamellar to hexagonal was not observed in our case [18], however, the identified phase is in good agreement with the phase diagram [28].

### 3.3. 5-FU Drug Releasing of the Pluronic Biomedical Hydrogels

**Figure 5:**
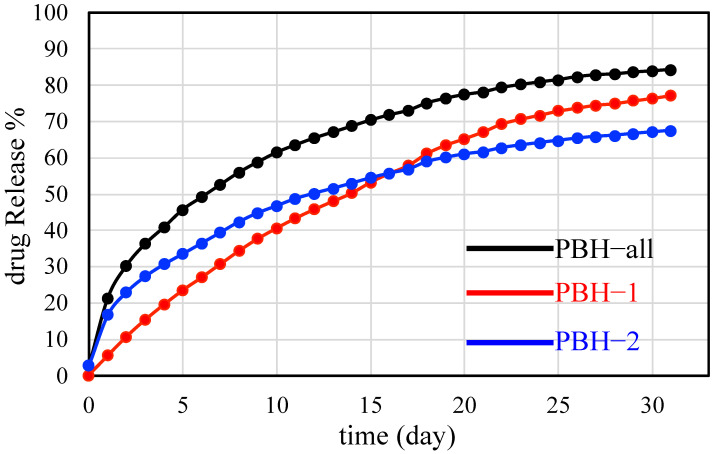
Drug releasing profiles of pluronic biomedical hydrogels. The experiment was contiguously monitored for one month.

In vitro release behavior of 5-FU from PBHs was assessed in an aqueous PBS medium (pH 7.4, containing 0.1% Tween 20 (*v*/*v*)) at 37 °C. At selected time intervals, release medium was withdrawn, replaced with an equal volume of fresh medium and analyzed in order to ensure sink conditions and to avoid precipitation. Results are reported as mean release percentage over time (*n* = 3) ± SD. All drug releasing profiles of the experimental PBHs with 5-FU loading were expressed at 37 °C for the purpose of simulating a microenvironment for human cellular uptake. Figure 5 demonstrates the monitoring of 5-FU drug release lasting for more than one month. Obviously, PBH-all gives the fastest 5-FU drug release among all the produced PBHs. PBH-1 has the lowest 5-FU drug release profile before day 16 and then accelerates to 77% at day 31. Although PBH-2 has a faster 5-FU drug release profile than PBH-1 before day 16, the continuous 5-FU drug releasing profile of PBH-2 goes below PBH-1 to 67% until the end of the experiment. This particular prolonged drug release phenomenon is interesting and rarely seen since general drug releasing is fast and released totally within one week. The cause relates to detailed structural differences.

## 4. Discussion

**Figure 6:**
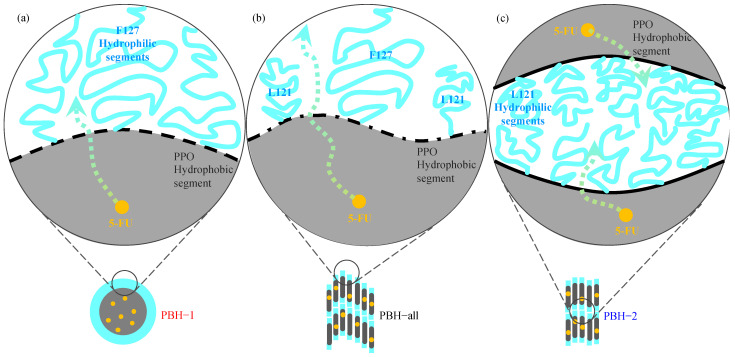
Microenvironment model of pluronic biomedical hydrogels. (**a**) PBH-1, (**b**) PBH-all, and (**c**) PBH-2.

Pluronic biomedical hydrogels (PBHs) were formulated in this study as a microenvironment to discover the drug delivering mechanism with respect to the migration of the anti-cancer drug 5-FU. Since PBHs have the same ingredient composition, the drug loading sequence can be differentiated to uncover the potential diffusion pathway across the material’s boundary. In this case, the material’s boundary comes from the self-assembly of pluronic copolymers with intrinsic amphiphilic segments. In PBH-1, 5-FU mainly stayed in the F127 micelle giving an enhanced FCC stacking structure according to the identified scattering peaks of Figure 4a. Similarly, 5-FU was concentrated in the L121 lamellar structure of PBH-2. The corresponding scattering peaks in the relative positions of 1:2 can be clearly seen in Figure 4b. The accompanying drug release profiles of Figure 5 demonstrate the significant influence of the self-assembled microstructure. PBH-all gives the fastest drug releasing profile beyond PBH-1 and PBH-2. Figure 6 depicts three possible physical models to explain the microenvironment of the PBHs as well as the drug releasing rate. As noted in Figure 6a of PBH-1, 5-FU stayed in the core of the F127 micelle and started to migrate across the amphiphilic boundary. The amphiphilic boundary is quite smooth, which allows 5-FU to leak out at some weak hydrophilic chain entanglements at around 100 ethylene oxide segments (i.e., the hydrophilic shell of micelle). On the other hand, 5-FU was embedded in the lamellar structure of PBH-2 as shown in Figure 6c. Its amphiphilic boundary was considered much flatter than that of PBH-1 in the curvature. Thus, a slower drug release trend should be observed for PBH-2 because 5-FU migrates across a stronger amphiphilic boundary (i.e., wall) resulting from a dense entangled hydrophilic chain. In the snapshot in Figure 6b of PBH-all, there is no doubt that the pluronic copolymers of F127 and L121 co-assemble if the hydrophobic segment lengths are compatible with each other to drive the self-assembly process [29]. It is noted that the spatial balance between the short hydrophilic segment of L121 and longer hydrophilic segment of F127 causes the undulated amphiphilic boundary. 5-FU instinctively to travels across the weaker amphiphilic boundary, which creates the fastest drug release curve of PBH-all. The microenvironment model of the PBHs in Figure 6 not only explains the kinetic migration of 5-FU but also shows more about the amphiphilic drug delivery vehicle compared to the lipid nanoparticle. On the other hand, a mass-fractal model was proposed by Prof. Vollet et al. to envision the domain structure of ambient-pressure drying silica aerogels between 2.8 to 4.0 nm [30]. It is a delicate model system to understand the aerogel specimen but PBHs are hydrogels with determined micelle or lamellar structures above a critical micelle concentration/temperature. Form factor analysis of SAXS is ongoing to disclose the detailed domain/form structural information with or without the addition of 5-FU at diluted concentrations. Recently, binary mixing of F127/L121 has gained some attention due to its optimized formulation in a high concertation or powder form [31]. There is a strong demand to manufacture a structurally stable and prolonged release drug carrier. Hopefully, the PBHs in this study could demonstrate a practical tool to improve the medical care of patients and even the possibility of a dual anti-cancer drug vehicle in the near future.

## 5. Conclusions

PBHs provide a microenvironment and a drug delivery system offering many attractive benefits such as intrinsic biocompatibility, ease of preparation, large-scale production and targeted delivery for clinical and practical applications. The main achievement of this study is to identify the drug release profiles along with the travel of 5-FU in pluronic biomedical hydrogels (PBHs) which could be altered via the preparation sequence even if all the ingredients are the same. The only difference is the 5-FU loading location in the pluronic copolymer either in a micelle structure or lamellar structure. PBH-2 presents the slowest result of prolonged release up to one month at 67%, which has rarely been seen before. The utilization of SAXS clearly and respectively demonstrates the micelle structure of F127, the lamellar structure of L121 and lamellar-like structure of the co-assembly of F127 and L121. The curvature of the amphiphilic boundary interacts with the interfacial movement of 5-FU, which explains the distinct drug releasing profiles in the aggregation of the self-assembled structure. In summary, the microenvironment model would make progress in disclosing the operating mechanism of anti-cancer drugs so as to adjust coordinated drug releasing if the ingredient composition is the same. Furthermore, the established microenvironment may mimic the cellular uptake or expulsion of incorporated chemotherapeutic drugs from the lipid bilayer.

## Figures and Tables

**Table 1:** The composition in volume (μL) of the pluronic biomedical hydrogels (PBHs).

Sample Code	F127 in H_2_O (25 wt%)	L121 in H_2_O (100 wt%)	5-FU in DMSO (0.2 M)
PBH-all	100	300	24
PBH-1	100 ^1^	300	24
PBH-2	100	300 ^2^	24

^1^ F127 was mixed with 5-FU first and then the complex was ultrasonically blended with L121. ^2^ L121 was mixed with 5-FU first and then the complex was ultrasonically blended with F127.

## Data Availability

Data is contained within the article.
